# Privacy and confidentiality measures in genetic testing and counselling: arguing on genetic exceptionalism again?

**DOI:** 10.1007/s13353-016-0339-4

**Published:** 2016-02-17

**Authors:** Magdalena M. Witt, Michał P. Witt

**Affiliations:** 1 Department of Rescue and Disaster Medicine, University of Medical Sciences, Poznań, Poland; 2 Department of Molecular and Clinical Genetics, Institute of Human Genetics PAS, Poznań, Poland

**Keywords:** Confidentiality, genetic counseling, privacy

## Abstract

Medical confidentiality in clinical genetics poses an important question about its scope, which would be in line with professional ethics and simple honesty. It is already known that the maintenance of absolute anonymity, bearing in mind the current progress of genetic techniques, is virtually impossible. On the other hand, our insight into the information contained in the human genome is increasing. This mini-review presents the authors’ standpoint regarding this complex and difficult issue.

The rapid development of modern techniques of human genome analysis, for both research and diagnostic purposes, entails a wide variety of problems, which have fallen and remain far behind the question of technological capabilities. These problems arise in the realm of social, ethical, religious and legal issues, and cover such issues as testing children for adult-onset disorders, incidental findings as a by-product of genetic testing, increased availability of direct-to-consumer genetic tests, patenting of genetic information, stratification of whole genome sequencing results, to name but a few (Berliner 2015).

Without the need to adopt a position in the long-term dispute on genetic data exceptionalism, it should be noted that there are certain features of genetic information differentiating it from other medical data, which raise strong objections. Since genetic traits run in families, they usually significantly impair the everyday life of many (if not all) family members. Their permanency and chronicity leads to psychological fatalism. They also cause discrimination and social stigma; the insight into future health status they provide causes a sense of insecurity affecting any aspect of life. Complexity and rare occurrence, the probabilistic character of results and the unknown real meaning of certain data currently uninterpretable further add to the burden (Bennett 2010).

Genomic population research and genetic testing, frequently based on large biobank repositories and accompanying databases, require sensible matching of genomic data with phenotypic descriptions; in many cases, the latter accelerates the potential to identify donors of biological materials. It has been shown that even small pieces of genetic information (SNPs data, expression or microbiome data) combined with publically available information, like surnames or parts of a postal address, make genomic research potentially identifiable (Gymrek et al. 2013). Thus, the common reassuring claim that the genetic analysis/research planned is anonymous proves to be inherently untrue. This emphasises the importance of the proper use of informed consent as the main tool for protecting the autonomy of the patient (Fiore and Goodman 2016).

This only partially demonstrates the difficulty of using genetic data for research and counselling. On the one hand, these data require increased security appropriate for sensitive personal data, while on the other, they bring the danger of their confidentiality being breached relatively easily. In genetic clinical practice, the extent of confidentiality (not to mention the ever-sensitive issue of accidental identification of non-paternity) frequently depends on the mode of inheritance of the trait studied.

In the case of recessive autosomal disorders, the identification of heterozygotes among the patient’s siblings can usually be relatively easily withheld during the process of counselling. The decision to test should be suspended until children are psychologically ready to make this decision independently. Psychological or social factors (e.g. extreme maternal anxiety) may justify exceptions to this principle. The release of information to other members of the family should remain in the hands of an experienced counsellor; however, a stand of the index case patient’s family is to be considered first. In case of a persistent controversy between the patient and the counsellor regarding whether the information should be disclosed to other members of the family, the standpoint of the local Ethical Commission should be considered.

Diagnostics/research of autosomal dominant traits, especially of neurodegenerative diseases of late onset, place a heavy burden on both the patient’s family and the counsellor. Pre-symptomatic and predictive testing of the children of an affected individual may cause elevated anxiety and fear of developing disease symptoms in the unforeseeable future: 50 % of the genetic risk might turn to certainty. However, no clues can be given as to the timeframe and severity of pathological symptoms. On the other hand, disclosure of pre-symptomatic genetic test results may assure the improbability of disease symptoms developing in persons with a 0 % genetic risk. Both situations create a completely different psychological context for the patient and his/her family. In this case, disclosure of relevant information to a third party might play an important role in warning other members of the family about serious pathological consequences still to emerge. It is hard to imagine this information being kept secret by a medical professional, even when the index case person/family insists on this. However, extreme caution and flexibility should be exercised by a genetic counsellor, reflecting the particular significance of ‘the right not to know’ for this group of families at risk (Bortolotti 2013). This requires precise qualification of whether we investigate the right to request non-disclosure of the information or a deliberate resignation (e.g. the patient does not set up an appointment with the doctor). Another issue is the patient’s right to request the destruction of the test results, which, as part of their medical records, do not remain in the hands of the patient and, in principle, are subject to administrative regulations.

The questions raised above lead clearly toward informed consent as a key tool for protecting patient autonomy (Appelbaum et al. 2014). The patient should know the subject of the consent in order to express their approval of physician’s actions or otherwise, which compensates for the difference in the doctor’s and the patient’s knowledge, at the same time, making the patient jointly responsible for the decisions taken (European Bioethical Convention 1997). Sensible planning of the diagnostic/research process based on family-derived material, a routine action in genetics, requires consideration of multiple scenarios which might occur as a result of the procedure. They should be included in the consent form and clearly explained to the patient/family in the pre-test phase at the beginning of the counselling process. When it is impossible to foresee and describe all options, an open alternative should be guaranteed, allowing the counsellor some freedom in adjusting his attitude to the situation. A shift from single-level communication (counsellor–patient) to multi-level (counsellor–family) should be considered quite early on. However, a binding decision on whether to inform at all, which relatives to inform etc. should be, rather, the result of a thorough discussion with the index case patient/immediate family at the very final stage of the diagnostic/counselling process, when all the details of the genetic diagnosis become clear. Ungoverned disclosure could be considered exclusively in the case of serious medical problems with significant prevention and treatability potential (Royal College of Physicians, The Royal College of Pathologists, The British Society of Human Genetics 2011). The quality of the communication process itself, in terms of the skills of the counsellor to convey information and the message clearly, might have a significant influence on the final result of counselling, including the decision regarding the possible disclosure of information to third parties. A starting point to all such decisions is a proper understanding of the problem by a patient/immediate family (d’ Audiffret Van Haecke and de Montgolfier 2015).

Is the patient lawfully entitled to decide whether to transfer information to a wider range of stakeholders or not; can such information be kept confidential even if it relates to the future risk of relatives developing the disease? This can be considered a conflict of particular rights and values: the patient’s right to privacy on the one hand and their family’s right to information when its concealment could entail a danger to their life or health on the other. Could a doctor depart from medical confidentiality in such a case? The question could also be put in a different way: is the result of a genetic test, and also the way in which it is conducted, a matter for the patient, or perhaps for the patient AND his/her family? In other words, who is the subject of the actions of a counsellor: the patient alone or the family at risk?

All these activities are based on the assumption of the indisputably ethical and lawful actions of the medical professional serving in a diagnostic/counselling process and/or researcher performing his/her duty and releasing medically significant results that are a by-product of the research activity. This is directly linked to the principles of beneficence (doing good), nonmaleficence (avoiding harm) and justice (burdens and benefits to be distributed fairly), which have to be fulfilled in all such activities both by family members and by a medical service providers/researchers (Berliner 2015). Adherence to these principles seems to be relatively easier for a service provider: lawful and ethical actions should, theoretically, enable this almost automatically. An assumption of such rules, however, can be misleading: legal regulations covering the field of medical genetics are not equally distributed among different countries, which makes the matter of ‘doing right’ in genetic counselling not obvious and straightforward. A basis for beneficence and nonmaleficence is even more obscure in family relations. It is usually the case that the law does not force anybody to aid others, as long as they are not hurt (with obvious exceptions).

However, one needs to remember that kinship relations entail duties regardless of whether we like it or not. There are enforceable duties of parents towards children and vice versa; people have moral duties to multiple family members, even if these people are severely disliked or even hated, just exclusively due to an unintended share in the gene pool (Berliner 2015). It would not be ethical to abandon this awareness when important medical facts of utmost significance for the health status of family members become available. In the Polish legal system, familial disclosure of genetic results remains ungoverned by any procedural norms. Similar situations exist in many countries; however, the Polish legal system seems to be unprepared for solving such complex problems due to the lack of a basic, consistent legal framework for medical practice in genetics. A draft of the relevant normative act still remains to be enacted.

The question of how strict one should be with the issue of confidentiality in genetics cannot be answered in simple terms. The confidentiality of medical details in all medical activities is a basis for patients’ trust and confidence (Kent 2003). Furthermore, genetic research is based on the trust of potential donors. In many cases, especially regarding rare diseases, keeping genetic data confidential with no option of returning the results to the patient/family would make it totally impossible to achieve the objectives of the study. Thus, due to the specific nature of genetic data, one needs to accept that, in genetics, confidence is not an absolute value and must be subjected to individual treatment. Furthermore, not all genetic data are of the same prognostic calibre, so, again, a case-by-case approach should be applied wisely. Does awareness of these facts add yet another argument in favour of genetic exceptionalism?
